# Enhanced Migratory Capacity of T Lymphocytes in Severe Chagasic Patients Is Correlated With VLA-4 and TNF-α Expression

**DOI:** 10.3389/fcimb.2021.713150

**Published:** 2021-11-02

**Authors:** Luiz Ricardo Berbert, Florencia Belén González, Silvina Raquel Villar, Carlos Vigliano, Susana Lioi, Juan Beloscar, Oscar Adelmo Bottasso, Suse Dayse Silva-Barbosa, Wilson Savino, Ana Rosa Pérez

**Affiliations:** ^1^ Laboratory on Thymus Research, Oswaldo Cruz Institute, Oswaldo Cruz Foundation, Rio de Janeiro, Brazil; ^2^ Institute of Clinical and Experimental Immunology, Faculty of Medicine, National University of Rosario and CONICET, Rosario, Argentina; ^3^ Department of Pathology, Favaloro Foundation, Buenos Aires, Argentina; ^4^ Cardiology Unit, Centenary Hospital and National University of Rosario, Rosario, Argentina; ^5^ Brazilian National Institute of Science and Technology on Neuroimmunomodulation, Oswaldo Cruz Institute, Oswaldo Cruz Foundation, Rio de Janeiro, Brazil; ^6^ Rio de Janeiro Research Network on Neuroinflammation, Oswaldo Cruz Institute, Oswaldo Cruz Foundation, Rio de Janeiro, Brazil

**Keywords:** lymphocyte migration, chronic chagasic myocarditis, VLA-4, cortisol, chemokines

## Abstract

*Trypanosoma cruzi* infection in humans leads to progression to chronic chagasic myocarditis (CCM) in 30% of infected individuals, paralleling T cell inflammatory infiltrates in the heart tissue. T-cell trafficking into the hearts of CCM patients may be modulated by *in situ* expression of chemotactic or haptotactic molecules, as the chemokine CXCL12, the cytokine tumor necrosis factor-alpha (TNF-α), and extracellular matrix proteins (ECM), such as fibronectin. Herein we evaluated the expression of fibronectin, CXCL12, and TNF-α in the myocardial tissue of *T. cruzi* seropositive (asymptomatic or with CCM), as well as seronegative individuals as healthy controls. Hearts from CCM patients exhibited enhanced expression of these three molecules. CXCL12 and TNF-α serum levels were also increased in CCM individuals. We then evaluated T lymphocytes from chronic chagasic patients by cytofluorometry, in terms of membrane expression levels of molecules involved in cell activation and cell migration, respectively, HLA-DR and the VLA-4 (very late antigen-4, being one integrin-type fibronectin receptor). Indeed, the expression of HLA-DR and VLA-4 was enhanced on T lymphocytes from chagasic patients, especially in the CCM group. To further approach the dynamics of T cell migratory events, we performed fibronectin-, TNF-α-, and CXCL12-driven migration. Peripheral blood mononuclear cells (PBMCs) and T cells from CCM patients presented an *ex vivo* enhanced migratory capacity driven by fibronectin alone when this ECM protein was placed in the membrane of transwell migration chambers. When TNF-α was previously placed upon fibronectin, we observed a further and significant increase in the migratory response of both PBMCs and T lymphocytes. Overall, these data suggest the existence in patients with chronic Chagas disease of a cardiac inflammatory infiltrate vector that promotes the recruitment and accumulation of activated T cells, driven in part by enhanced tissue expression of fibronectin and TNF-α, as well as the respective corresponding VLA-4 and TNF receptors.

## Introduction

Chagas disease is the main parasitic disease in Latin America, being caused by infection with the protozoan parasite *Trypanosoma cruzi*. It is estimated that ~30% of *T. cruzi*–infected individuals progress to chronic and irreversible disorders, where chronic chagasic myocarditis (CCM) is the most frequent clinical manifestation (Rassi et al., 2010). Despite that Chagas disease etiopathogenesis remains controversial, a bulk of evidence supports the hypothesis that *T. cruzi* may persist in the host during several years, stimulating the development of a chronic inflammatory response that results in tissue damage (Tarleton, 2001). Yet, the simultaneous existence of autoimmune reactions cannot be ruled out (Bonney and Engman, 2015).

In recent times, much effort has been made to identify immunological factors and molecular mechanisms involved in the development of CCM. Among them, the recruitment of activated T lymphocytes and the accumulation of these cells within the cardiac tissue are believed to play a pivotal role in the pathogenesis of the disease, favoring a proinflammatory microenvironment compatible with chronic inflammatory myocarditis (Vitelli-Avelar et al., 2006; Silverio et al., 2010). In addition, we recently showed that CCM is associated to persistently increased levels of immune and metabolic proinflammatory mediators, along with an adverse endocrine anti-inflammatory response that may contribute to the underlying mechanisms related to myocardial tissue damage (González et al., 2018).

During *T. cruzi* infection, the recruitment and migration of activated T lymphocytes to the heart seem to be key steps to contain parasitism but also contributing to tissue damage. Both processes are complex and not only depend on the state of activation of T lymphocytes but also involve proinflammatory cytokines, chemokines, adhesion molecules, and extracellular matrix (ECM) constituents and corresponding receptors.

Integrins are cell surface receptors capable of mediating cell–cell and cell–matrix contacts. The integrin very late antigen-4 (VLA-4) has a key role in the cellular immune response since it mediates the recruitment of leukocytes to sites of inflammation and also bind to ECM components. The vascular cell adhesion molecule-1 (VCAM-1) and fibronectin are the two well-characterized interaction partners of VLA-4 (Schlesinger and Bendas, 2015). In the context of *T. cruzi* infection, some findings from animal models proposed that VCAM-1 expression on vascular endothelial cells may have an essential role in T cell attraction to the heart, with recruitment of peripherally activated VLA-4^+^CD8^+^ T lymphocytes (dos Santos et al., 2001). Additionally, VLA-4 expression in infiltrating T cells may contribute to T cell attachment to ECM, particularly fibronectin, resulting in a prevalence of this cell population in the inflamed heart. Moreover, VLA-4/fibronectin interactions may stimulate their effector functions and consequently prompt myocarditis (dos Santos et al., 2001). Previous studies on immature T lymphocytes from *T. cruzi*–infected animals showed an enhancement of their migratory capacity when fibronectin acted as a haptotatic stimulus in combination with the chemokine CXCL12 (Mendes-da-Cruz et al., 2006).

In addition, the proinflammatory cytokine TNF-α plays a key role in controlling parasite load during acute *T. cruzi* infection (Lima et al., 1997; Castaños-Velez et al., 1998; Lannes-Vieira et al., 2011). However, the presence of TNF-α in the myocardium of chronically infected mice and humans suggests that besides controlling tissue parasitism, they may contribute directly or indirectly to the recruitment of inflammatory cells and the establishment of myocarditis (Reis et al., 1993; Higuchi et al., 1997; Talvani et al., 2000). We previously showed that TNF-α can modulate the *ex vivo* fibronectin-driven migration of immature T cell precursors during experimental *T. cruzi* infection and can influence the severity of myocarditis (Pérez et al., 2007; Pérez et al., 2009; Pérez et al., 2012).

Aiming to understand more clearly the dynamics of T-cell migratory events that may influence the development of CCM, we evaluated herein the migratory capacity of T cells derived either from healthy or chronic chagasic patients, with or without cardiac involvement, and whether such migratory response is influenced by chemotactic, e.g., CXCL12, TNF-α, or haptotactic molecules such as the ECM protein fibronectin.

## Material and Methods

### Study Population

The population in which the migratory responses of T lymphocytes were evaluated consisted of 46 individuals with *T. cruzi* chronic infection and 20 age-matched healthy volunteers recruited at the Chagas Disease Service from the Department of Cardiology, Hospital Provincial del Centenario de Rosario, National University of Rosario (Rosario, Argentina). The diagnosis of Chagas disease was based on at least two standard serological tests (including ELISA, indirect immunofluorescence, and/or hemagglutination), together with clinical symptoms, heart/chest X-ray, and 12-lead resting electrocardiogram (ECG). None of these patients received parasite-specific treatment (i.e., Benznidazole or Nifurtimox) or had concomitant pathological disorders. Exclusion criteria comprised other infections, neuroendocrine disturbances, immunological diseases, or treatment with hormones or immunomodulators. Control subjects were consistently seronegative to *T. cruzi*–specific tests.

Patients with chronic Chagas disease were grouped according to their degree of cardiac involvement, as follows: Asymptomatic group (n=20), with normal ECG and chest X-ray and without symptoms, and Cardiac group (n=26). In some analyses, the Cardiac group was further split into a subgroup with mild cardiac involvement (Mild group, n=13). In this subgroup, patients showed any of the following alterations: incomplete right bundle branch block or complete right bundle branch block, ventricular arrhythmia, and chest X-ray cardiothoracic ratio <0.55 but without congestive heart failure. The second subgroup was characterized by severe myocarditis (Severe group, n=13), bearing congestive heart failure, pathological ECG profiles, and/or heart enlargement detected by chest X-ray (cardiothoracic ratio >0.55). All participants provided written informed consent to protocols used here and approved by the local Ethical Committee of the Medical Faculty of National University of Rosario (Resolution n° 2854/2008). The main characteristics of the studied population are summarized in **Supplementary Table 1**.

### Histology and Immunofluorescence of Cardiac Tissue

Heart tissue samples from cardiac transplanted patients bearing chronic Chagas disease were collected at Favaloro Hospital, Buenos Aires. Heart control samples were obtained from cardiac transplanted individuals serologically negative for *T. cruzi*. All participants provided written informed consent to protocols used here and approved by the Fundación Favaloro Ethical Committee (Res. N° 605/16). Histological analysis of inflammatory infiltrates was performed on paraffin sections stained with hematoxylin-eosin. To analyze the expression of fibronectin, CXCL12, and TNF-α, tissue samples were deparaffinized and hydrated in the sequence of two baths of xylene absolute ethanol 95% and 70% distilled water, followed by PBS. Slides were then immersed in sodium citrate buffer (10 mM, pH 6.0) and inserted in the microwave at full power (three cycles of 5 min each) followed by a PBS bath. Possible interactions with Fc receptors were blocked with incubation of the samples with PBS/BSA/2% goat serum for 1 h. Slides were subjected overnight to optimal concentrations of primary antibodies specifically recognizing fibronectin (DAKO Co., USA), CXCL12 (Santa Cruz, USA), and human TNF-α (Abcam, UK). After PBS washing, we applied appropriate Alexa-488 fluorochrome-labeled secondary antibodies (Invitrogen, USA) for 45 min. Settings were adjusted using an isotype control antibody (Santa Cruz, USA). Following the immunostaining, the specimens were washed in PBS and mounted on coverslips with mounting medium containing antifading reagent (ProLong, Invitrogen, MA, USA) and analyzed with a fluorescence microscope Zeiss Axio Imager A2 and Axiovision Release software (USA). The quantification of the mean fluorescence intensity was determined using ImageJ software, through analysis of five random fields for each marker.

### Cytofluorometry

Peripheral blood mononuclear cells (PBMCs) were obtained from fresh EDTA-treated blood samples. The blood was layered over a Ficoll-Paque-PLUS (GE-Healthcare, IL, USA) gradient and centrifuged at 400 g for 30 min at 25°C. The buffy coat was washed and resuspended in RPMI 1640 medium. PBMCs thus obtained were stained by monoclonal antibodies conjugated with fluorescein isothiocyanate (FITC), phycoerythrin (PE), phycoerythrin coupled to the cyanine dye Cy5TM (PE Cy5), peridinin-chlorophyll-protein (PerCP), or allophycocyanin (APC) for the following cell surface markers: CD3, CD4, CD8, HLA-DR, and CD49d (alpha-4 integrin chain); all from BD Bioscience (CA, USA). Before adding antibodies, the Fc receptor was blocked with total human AB^+^ serum (Sigma-Aldrich, MO, USA) for 15 min. Cells were then incubated with the antibodies in the dark for 30 min at 4°C. For flow cytometry analysis, cells were gated based on their forward *versus* side scatter profiles, and data were collected using a FACSCalibur or FACSAria device. Data analysis was performed using the FlowJo software (all from BD Bioscience, CA, USA).

### *Ex Vivo* Cell Migration Assays

*Ex vivo* cell migration assays were performed using transwell chambers (Corning Costar, Cambridge MA, USA). Aliquots of one million isolated PBMCs were placed onto 5 µm pore *transwell* inserts previously coated on both sides with fibronectin (10 µg/ml) for 1 h and then blocked on both sides with BSA (10 µg/ml) for 45 min. In a second group of experiments, we added or not different stimuli: CXCL12 (400 ng/ml) or TNF-α (250 pg/ml) 15 min before migration over fibronectin-coated upper chamber. For migration assays, 1.10^6^ cells were placed in the upper chamber of transwell inserts and incubated in RPMI medium with 1% BSA at 37°C in 5% CO_2_ for 4 h. Cells that migrated towards the lower compartment were collected, counted, and analyzed by flow cytometry after staining with the same markers described above. Assays were conducted in duplicates and compared with control wells that contained medium with BSA alone. For migration over fibronectin, results were reported as the percentage of input: % Input = (absolute number of migrating cells with a given phenotype/Total number of starting cells with the same given phenotype) × 100. For cell migration driven by CXCL12 or TNF-α, results are shown as Δ Input = (% Input with TNF-α or CXCL12) − (% Input over fibronectin).

### Statistical Analysis

Comparisons among all groups were done by applying the Kruskall-Wallis non-parametric analysis of variance followed by the Mann-Whitney U test. Results were expressed as mean ± standard error (SE) unless otherwise indicated. The GraphPad InStat 6.0 software (GraphPad, CA, USA) was applied for statistical analyses, and differences were considered significant when the *p* value was ≤0.05.

## Results

### Chagasic Cardiomyopathy Correlates With Enhanced Expression of Fibronectin, CXCL12, and TNF-α in Myocardial Tissue and Higher Circulating Levels of CXCL12 and TNF-α

**Figure 1:**
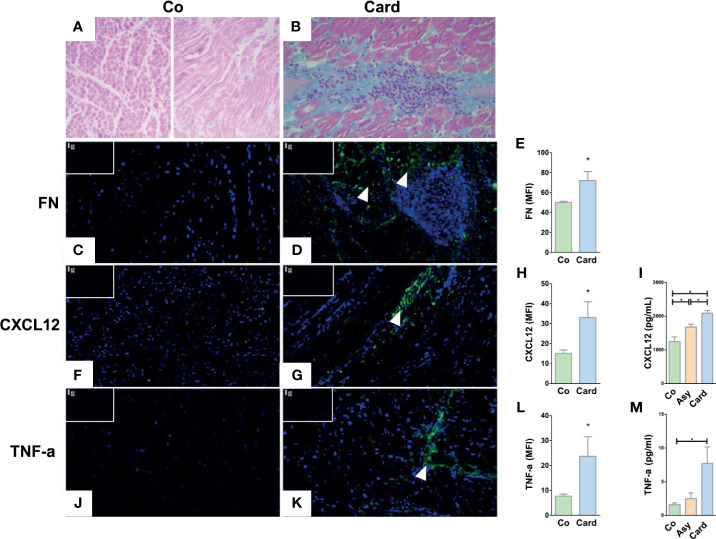
Histological and immunofluorescence analyses of the cardiac tissue of Control subjects and *T. cruzi*–infected patients with chronic myocarditis. Hematoxylin and eosin staining of healthy myocardium from Control individuals **(A)** and from Cardiac patients **(B)** showing the typical diffuse inflammatory infiltrate of *T. cruzi* chronic infection associated with the inflammatory cell influx. Immunofluorescence images reveals an increased fibronectin (FN) deposition (fibronectin in green, cell nuclei in blue) in the heart sections from Cardiac patients compared to Controls **(C, D)**. The mean fluorescence intensity (MFI) of fibronectin was also increased in the Cardiac group **(E)**. CXCL12 immunolabeling (CXCL12 in green, cell nuclei in blue) showed an augmented expression **(F, G)** and MFI **(H)** in Cardiac patients compared to Controls. Systemic amounts of CXCL12 are also enhanced in *T. cruzi*–infected individuals, despite being Asymptomatic or Cardiac **(I)**. Immunofluorescence images of heart sections from Cardiac patients showing an increased TNF-α expression (TNF-α in green, cell nuclei in blue) **(J, K)** and MFI **(L)** compared to Controls. Systemic amounts of TNF-α are also enhanced in Cardiac individuals **(M)**. Isotype controls are shown in the upper left corner of each image. In all cases, MFI was evaluated by measuring five images/heart per study group (Control, n = 3 and Cardiac, n = 3). *p < 0,05 *versus* Controls. Mann-Whitney U test was used for statistical analyses. Co, Control; Asy, Asymptomatic; Card, Cardiac.

T cell trafficking process into the heart of infected patients may be modulated by *in situ* expression of chemotactic or haptotactic molecules, like ECM proteins and immune factors. In this context, we first performed a histological analysis and immunofluorescence detection of fibronectin, CXCL12, and TNF-α in myocardial tissues obtained from explanted hearts from non-chagasic individuals (Control group) and from *T. cruzi*–infected patients with chronic myocarditis (Cardiac group) that underwent cardiac transplantation. Compared to Controls (**Figure 1A**), myocardial tissue sections from Cardiac patients exhibited obvious infiltrates and fibrosis (**Figure 1B**) and increased immunoreactivity for fibronectin (**Figures 1C–E**), CXCL12 (**Figures 1F–H**), and TNF-α (**Figures 1J–L**).

Moreover, CXCL12 circulating levels were higher in all *T. cruzi*–infected patients (both Asymptomatic and Cardiac), with the Cardiac group presenting the largest increase (**Figure 1I**). At the systemic level, TNF-α amounts were also increased in the Cardiac group compared with Controls (**Figure 1M**). Additional analysis also indicated TNF-α amounts were higher in Cardiac subjects with more severe pathology compared to those with mild carditis (Mild = 3.1 ± 1.8; Severe = 7.7 ± 2.4; p<0.05). Together, these data suggest that a systemic inflammatory profile and an increase in chemotactic/haptotatic stimuli driven by these factors might boost the recruitment of activated T lymphocytes into the cardiac tissue.

### The Expression of HLA-DR and VLA-4 on T Lymphocytes Positively Correlates With Chagas Disease Progression

**Figure 2:**
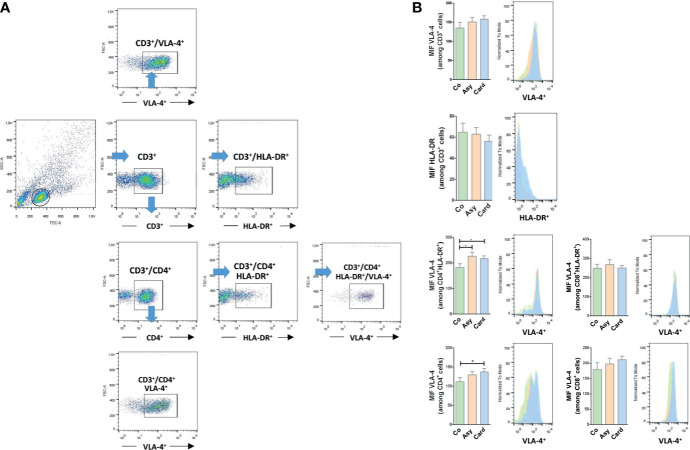
VLA-4 and HLA-DR surface expression in T lymphocytes from chronically *T. cruzi*–infected individuals. **(A)** Gating strategy for flow cytometry analysis. Cells were first gated on SSC-A *versus* FSC-A. The lymphocyte gate thus obtained was further analyzed to determine T cell proportions and their expression of HLA-DR and VLA-4 expression. **(B)** The median fluorescence intensity (MFI) of HLA-DR and VLA-4 were also evaluated in each subpopulation. *p < 0.05 *versus* Controls. Kruskal-Wallis followed by Mann-Whitney U test were used for statistical analyses. Co, Control (n = 20); Asy, Asymptomatic (n = 20); Card, Cardiac (n = 26).

To define whether we could find a correlation between the membrane expression profiling for HLA-DR and VLA-4 on T lymphocytes with disease progression, we evaluated by flow cytometry the proportion and surface density of each marker in peripheral T cells from Control, Chagasic Asymptomatic, and Cardiac patients. **Figure 2A** shows the gating strategy used for flow cytometry analysis. The proportions of the different subpopulations are showed in **Table 1**. While the percentage of circulating CD3^+^ T cells decreased in Asymptomatic and Cardiac compared to Control individuals, the relative numbers of CD3^+^ T cells expressing HLA-DR and VLA-4 increased, especially in Cardiac patients. **Table 1** also shows that CD4^+^ and CD8^+^ T cell activation is notorious as the severity of the pathology increases (Cardiac over Control HLA-DR fold-increase for CD8^+^ T cells ~3.2 and for CD4^+^ T cells ~4.1). Total CD8^+^ T cells as well as activated CD8^+^HLA-DR^+^ T cells showed a high expression of VLA-4, regardless of whether individuals were infected or not (**Table 1**). On the contrary, we found an increased expression of VLA-4 in CD4^+^ T cells from *T. cruzi*–infected patients, whereas its co-expression with HLD-DR is ~15% higher in infected than in Control individuals (**Table 1**). Furthermore, CD4^+^ T cells and CD4^+^HLA-DR^+^ T cells significantly increased the surface density of VLA-4, mainly in the Cardiac group (**Figure 2B**).

**Table 1:** Percentages of T cell subpopulations expressing VLA-4 and HLA-DR in Control, *versus* Asymptomatic and Cardiac chagasic groups.

Markers (%)	Control (n = 20)	Asymptomatic (n = 20)	Cardiac (n = 26)
**CD3^+^**	70.51 ± 1.986	62.22 ± 2.158^**^	62.64 ± 2.342^*^
**CD3^+^HLADR^+^**	7.437 ± 1.002	13.7 ± 1.797^**^	26.82 ± 2.704^****^
**CD3^+^VLA-4^+^**	87.81 ± 1.512	91.72 ± 1.63^*^	91.84 ± 0.7279^*^
**CD3^+^CD4^+^**	64.35 ± 2.467	66.09 ± 2.851	61.72 ± 3.732
**CD4^+^HLADR^+^**	3.916 ± 0.4157	9.216 ± 1.235^***^	16.02 ± 1.423^****^
**CD4^+^VLA-4 ^+^**	82.84 ± 1.506	88.96 ± 2.164^**^	87.26 ± 0.8967^*^
**VLA4^+^/CD4^+^HLADR^+^**	73.39 ± 2.862	88.78 ± 1.981^***^	88.23 ± 1.528^***^
**CD3^+^CD8^+^**	31.91 ± 2.118	28.62 ± 2.256	30.08 ± 2.795
**CD8^+^HLADR^+^**	13.49 ± 2.55	23.02 ± 2.575^*^	43.55 ± 3.021^****^
**CD8^+^VLA-4^+^**	95.99 ± 1.537	98.13 ± 0.696	98.89 ± 0.4381
**VLA4^+^/CD8^+^HLADR^+^**	97.1 ± 1.983	99.38 ± 0.1542	99.71 ± 0.1322

Values are expressed as mean ± SEM. *p < 0.05, **p < 0.01, and ***p < 0.005; ****p < 0.001 versus Control group

### Activated T Cells From Patients With Cardiomyopathy Exhibited Enhanced Fibronectin-Driven Migratory Response

**Figure 3:**
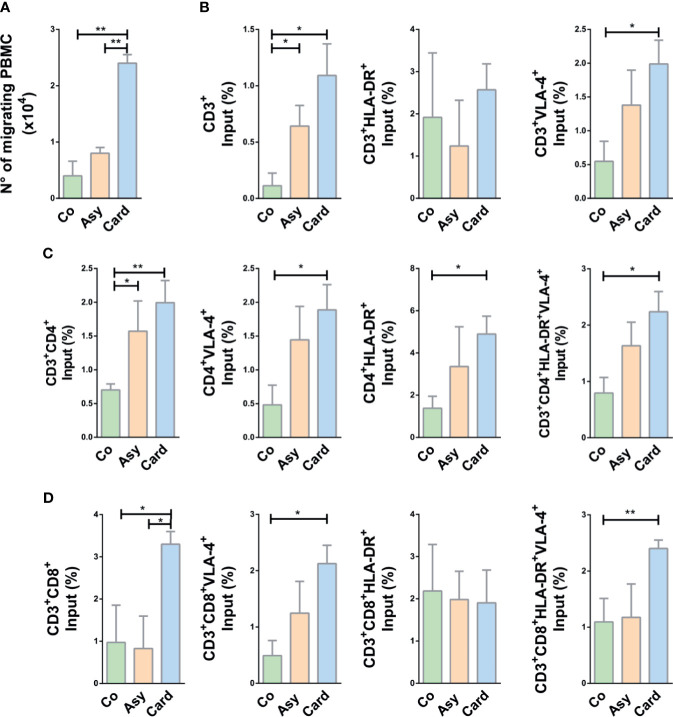
Migratory capacity of T cells from chagasic patients over fibronectin. **(A)** The graph shows the absolute number of migrating peripheral blood mononuclear cells (PBMC). **(B)** Percentage of input of CD3+ T cell subpopulations. **(C)** Percentage of input of CD4+ T cell subset. **(D)** Percentage of input of CD8+ subpopulations. In all cases, cells were allowed to migrate in *transwell* chambers coated with fibronectin or BSA. The values shown correspond to specific migration after subtracting numbers obtained for each sample in wells coated with BSA alone. *p < 0.05 and **p < 0.01 *versus* Controls. Kruskal-Wallis followed by Mann-Whitney U test were used for statistical analyses. Co, Control (n = 20); Asy, Asymptomatic (n = 20); Card, Cardiac (n = 26).

To evaluate the migratory potential of PBMCs and particularly T cells derived from chronically *T. cruzi*-infected subjects compared to cells from Control individuals, we performed *ex-vivo transwell* cell migration assays under the *stimuli* of fibronectin, CXCL12, and TNF-α, combined or not. First, we investigated the putative role of fibronectin upon T cell migration in Chagas disease. Since T cells from infected individuals might exhibit higher *ex vivo* cytokinesis than T cells from Control subjects, we systematically discounted the values recorded after migration in transwells covered with BSA alone. As shown in **Figure 3A**, PBMCs from infected subjects showed an enhanced migratory response compared to Controls, being significantly higher in the Cardiac group (~5-fold increase, as compared to Controls) when fibronectin was used as coating of *transwell* chambers without any other stimulus.

To evaluate activation of migrant T cells and correlate this pattern with the influx observed in *ex-vivo* migration, we analyzed the co-expression of HLA-DR^+^ and VLA4^+^ in T cells after migration. The input of CD3^+^ T cells favored by fibronectin is clearly enhanced in Cardiac patients, being this augmented motility positively correlated to VLA-4 expression but not to HLA-DR (**Figure 3B**). Total CD4^+^, CD4^+^VLA-4^+^, and CD4^+^HLA-DR^+^ T cells from Cardiac patients also exhibited enhanced motility, and cells from Asymptomatic subjects showed a similar tendency (**Figure 3C**). Migratory CD4^+^ T cells co-expressing VLA-4 and HLA-DR were also significantly augmented in Cardiac patients (**Figure 3C**). A similar pattern was observed for CD8^+^ T cells, with exception of CD8^+^HLA-DR^+^ T cells (**Figure 3D**).

### TNF-α but Not CXCL12 Enhanced the *Ex Vivo* Fibronectin-Driven Migratory Response of PBMCs and Activated T Cells From Cardiac Patients

**Figure 4:**
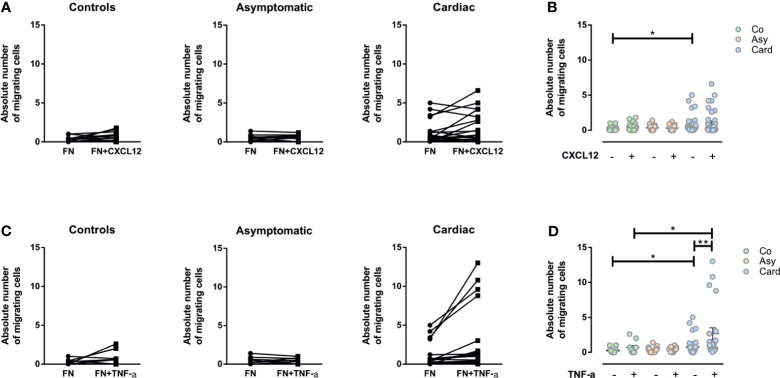
Evaluation of the migratory capacity of peripheral blood mononuclear cells (PBMCs) driven by CXCL12 or TNF-α. **(A)** Graphs show the absolute numbers of by migrating PBMCs from control, asymptomatic, and cardiac subjects in *transwell* chambers coated by fibronectin (FN) or FN+CXCL12. **(B)** The absolute number of migrating PBMCs in the different groups without and with CXCL12. **(C)** The absolute number of migrating PBMCs in *transwell* chambers coated with FN and FN+TNF-α from control, asymptomatic, and cardiac subjects. **(D)** The absolute number of migrating PBMCs in the different groups without and with TNF-α. In all cases, the values shown correspond to specific migration after subtracting numbers obtained for each sample in wells coated with BSA alone. *p < 0.05 and **p < 0.01 *versus* Controls. Kruskal-Wallis followed by Mann-Whitney U test were used for statistical analyses. Co, Control (n = 20); Asy, Asymptomatic (n = 20); Card, Cardiac (n = 26).

Considering that CXCL12 is strongly chemotactic for T lymphocytes, and since TNF-α could generate a significant enhancement in the migratory response of T cells when associated with other molecules as fibronectin, we evaluated if both molecules could increase the *ex vivo* fibronectin-driven migratory response of PBMCs and T cells. Although a tendency could be observed towards an increase in migratory response to CXCL12 in PBMCs from the Cardiac group, the differences seen were not statistically significant (**Figures 4A, B**). Strikingly, however, the addition of TNF-α significantly increased the fibronectin-driven migratory response of PBMCs from Cardiac patients (**Figures 4C, D**).

**Figure 5:**
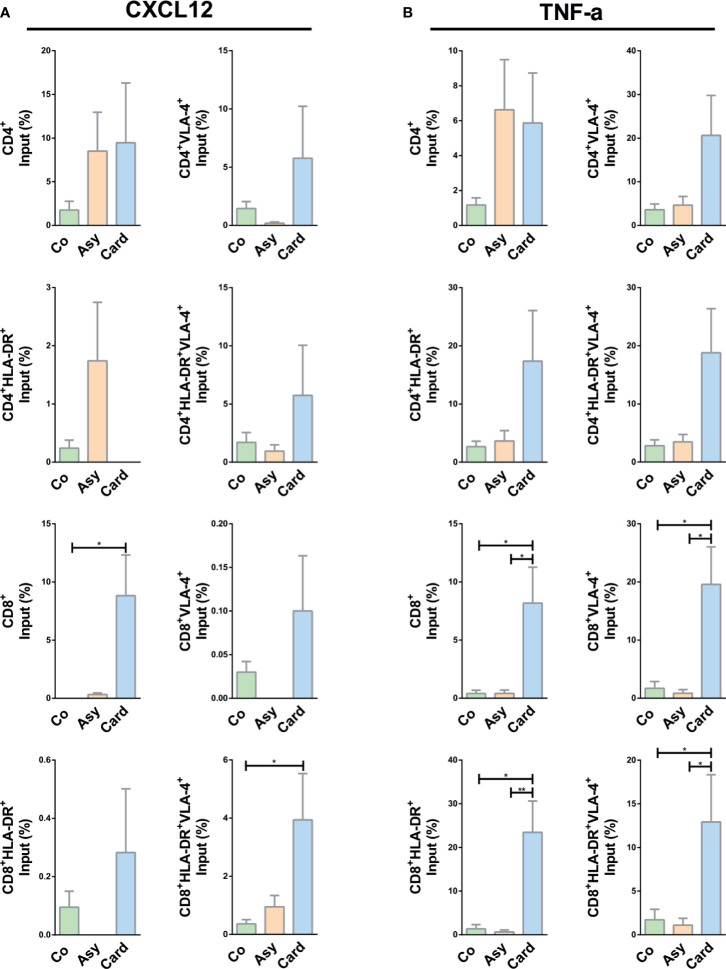
CXCL12- or TNF-α-driven migratory response of T cells from chagasic patients. **(A)** Percentage of input of different T cell subpopulations expressing VLA-4 and HLA-DR in FN+CXCL12 coated transwells. **(B)** Percentages of input for different T-cell subpopulations expressing VLA-4 and HLA-DR in FN+ TNF-α coated transwells. *p < 0.05 and **p < 0.01 *versus* Controls. Kruskal-Wallis followed by Mann-Whitney U test were used for statistical analyses. Co, Control (n = 20); Asy, Asymptomatic (n = 20); Card: Cardiac (n = 26).

We then assessed the migration of activated T cells under CXCL12 and TNF-α *stimuli*. While no changes were observed in CD4^+^ T cell subpopulations, total CD8^+^ and activated CD8^+^HLA-DR^+^VLA-4^+^ T cells from the Cardiac group migrated more in the presence of CXCL12 (**Figure 5A**).

Under TNF-α stimulus, activated CD4^+^ T cells (CD4^+^VLA-4^+^, CD4^+^HLA-DR^+^, CD4^+^HLA-DR^+^VLA-4^+^) from chronically *T. cruzi*–infected patients presented a tendency of an increased migratory activity (**Figure 5B**). In addition, all CD8^+^ T cells subpopulations from Cardiac patients exhibited an increased fibronectin-driven migratory response (**Figure 5B**), reinforcing the notion that molecular interactions mediated by fibronectin and TNF-α stimulate the influx of activated cytotoxic lymphocytes inside the myocardium, particularly CD8^+^HLA-DR^+^VLA-4^+^ T cells.

## Discussion

Chagasic cardiopathy is the most serious manifestation of chronic Chagas disease, where the contribution of T cells to heart tissue damage is evident. However, some aspects of CCM pathogenesis remained partially unclear, in particular regarding the molecular interactions involved in the T cell infiltration process. Herein, we provided *in situ* and *ex vivo* evidence indicating that T cell trafficking into the heart of patients with CCM may be supported by the *in situ* enhanced expression and/or deposition of fibronectin and TNF-α.

We found that serum and heart tissue from Cardiac patients exhibited a proinflammatory milieu enriched in TNF-α, as previously shown (Ferreira et al., 2003; Perez et al., 2011; Rodrigues et al., 2012). Moreover, increased fibronectin deposition was observed in the heart tissue of CCM individuals (Waghabi et al., 2009), thus in agreement with our present results. Similarly, within the myocardium of mice infected with *T. cruzi*, an enhancement of fibronectin and other ECM components was detected, overlapping with the accumulation of the inflammatory infiltrate (Pereira et al., 2014; Coelho et al., 2018). In the *transwell* migration assays, the combination of fibronectin with TNF-α induced an increase in the migratory response of lymphocytes from patients with Chagas disease, revealing the functional relationship between cytokines and ECM proteins such as fibronectin.

It has been reported that *T. cruzi*–infected humans and mice displayed an enhanced frequency of CD4^+^ and CD8^+^ cells within the intracardiac inflammatory infiltrates, with a predominance of CD8^+^ T cells being correlated with the progression of the cardiac disease (Higuchi M de et al., 1993; Tarleton et al., 1994; Fuenmayor et al., 2005). In the same vein, activated CD3^+^HLA-DR^+^ T effector cells were observed in the blood of Cardiac patients (Dutra et al., 1994; Lepletier et al., 2014). Herein, we also showed that Cardiac individuals displayed circulating HLA-DR^+^VLA-4^+^ T cells, thus compatible with an activated/memory phenotype (dos Santos et al., 2001) potentially being attracted by fibronectin alone or in combination with TNF-α, as shown by the *ex vivo* cell migration assays. Accordingly, PBMCs and T cells from Cardiac patients presented an enhanced migratory capacity driven by fibronectin, but not by CXCL12, despite the existence of a marked tendency, which is in line with previous studies using immature T cells from *T. cruzi*–infected mice, showing that the presence of CXCL12 increase the migratory capacity of thymocytes (Mendes-da-Cruz et al., 2006).

Based on the present and previous results (Mendes-da-Cruz et al., 2006; Pérez et al., 2012), we postulate that circulating HLA-DR^+^VLA-4^+^ T lymphocytes are involved in the development of a cardiac inflammatory infiltrate vector. Furthermore, under these conditions, it is conceivable that inflammatory cytokines such as IFN-γ or TNF-α induce in the infiltrating T lymphocytes an additional increase of HLA-DR, thereby improving the recognition of parasite-derived peptides or parasite-mimetic peptides displayed on the surface of the cardiomyocytes (Corrêa-Oliveira et al., 1999). Thereby, the association between VLA-4/fibronectin and VLA-4/fibronectin/TNF-α seems to be important for T-cell functional activation, as well as for the stimulation of their migratory capacity. Our results suggest that the presence of TNF-α and the enhanced deposition of fibronectin in the myocardium may contribute to the recruitment of inflammatory T cells, resulting in a complex chain of events that reinforce themselves through a feedback loop, ultimately favoring the development and establishment of carditis.

Lastly, since leukocyte influx towards target tissues is at least in part regulated by the interaction of cytokines and/or chemokines with ECM components, manipulating these interactions before the establishment of the cardiac damage may represent a potential therapeutic approach in chronic chagasic patients. Nevertheless, discordant results were reported in preclinical settings after blocking TNF-α (Bilate et al., 2007; Pérez et al., 2009; Pereira et al., 2014; Pereira et al., 2015), and the administration of an anti-TNF antibody to chronic chagasic patients correlated with Chagas disease reactivation (Vacas et al., 2017; Ringer et al., 2021), thus preventing its use in chagasic patients. By contrast, the administration of an anti-VLA-4 antibody attenuated experimental *T. cruzi*–driven brain inflammation, since it abrogated T-cell influx towards neuroendocrine tissues by blocking VLA-4/VCAM-1 and VLA-4/fibronectin interactions (Roffê et al., 2003). These findings indicate that such interaction can be envisioned as a therapeutic target aiming at blocking the influx of activated and pathogenic T cells to the heart.

## Data Availability Statement

The original contributions presented in the study are included in the article. Further inquiries can be directed to the corresponding authors.

## Ethics Statement

All participants provided written informed consent to protocols used here and approved by the local Ethical Committee of the Medical Faculty of National University of Rosario (Resolution n° 2854/2008). Heart control samples were obtained from cardiac transplanted individuals serologically negative for *T. cruzi*. All participants provided written informed consent to protocols used here and approved by the Fundación Favaloro Ethical Committee (Res. N° 605/16). The patients/participants provided their written informed consent to participate in this study.

## Author Contributions

LB and FG carried out the experiments, processed the experimental data, and wrote the manuscript. SV and SL assisted with human samples. CV, JB, and OB did patient recruitment, clinical evaluation, and cardiac tissue sampling. SS-B, WS, and AP contributed to the design and implementation of the research, to the analysis of the results, and to the writing of the manuscript. All authors discussed the results and commented on the manuscript. All authors contributed to the article and approved the submitted version.

## Funding

This work was supported by Fiocruz, CNPq, CAPES, and FAPERJ (Brazil), and the Mercosur Fund for Structural Convergence (FOCEM). The study was developed in the frameworks of the Brazilian National Institute of Science and Technology on Neuroimmunomodulation (CNPq) and the Rio de Janeiro Research Network on Neuroinflammation (Faperj). This work was further supported by grants provided by Argentinean funding institutions: CONICET (PIP 0789), ASACTeI (2010-049-12), and SECYT-UNR (1MED410).

## Conflict of Interest

The authors declare that the research was conducted in the absence of any commercial or financial relationships that could be construed as a potential conflict of interest.

## Publisher’s Note

All claims expressed in this article are solely those of the authors and do not necessarily represent those of their affiliated organizations, or those of the publisher, the editors and the reviewers. Any product that may be evaluated in this article, or claim that may be made by its manufacturer, is not guaranteed or endorsed by the publisher.
