# Supplementary material for: Enhanced Migratory Capacity of T Lymphocytes in Severe Chagasic Patients Is Correlated With VLA-4 and TNF-α Expression
Source: Front Cell Infect Microbiol. 2021 Nov 2;11:713150. doi: 10.3389/fcimb.2021.713150 (PMC8593233; doi:10.3389/fcimb.2021.713150)
Supplement: Supplementary file 1 [file Table_1.docx]

| **Groups** | **N° of individuals** | **Female/male ratio** | **Age range (years)**  **mean ± SEM** | **Clinical Findings** | **Thorax RX Findings** | **ECG Findings** |
| --- | --- | --- | --- | --- | --- | --- |
| **Control** | 20 | 9/11 | 49.9 ± 2.9 | w/s | Normal | Normal |
| **Asymptomatic** | 20 | 11/9 | 46.0 ± 3.0 | w/s | Normal | Normal |
| **Cardiac** | 26 | 16/10 | 53.1± 2.6 |  | | |
| ***Mild*** | 13 | 7/6 | 52.9 ± 2.4 | w/s | Normal | Altered |
| ***Severe*** | 13 | 9/4 | 54.0 ± 3.7 | Symptomatic | Heart Enlargement | Altered |

**Supplementary table 1. Main features of the study population.** w/s: without symptoms
